# Combined use of niraparib enhanced the inhibitory effect of Anti-GD2 antibody on osteosarcoma cells

**DOI:** 10.1007/s12672-024-01166-y

**Published:** 2024-07-24

**Authors:** Chen Wenyao, Ma Shuai, Fan Yifeng, Li Xinzhi, Que Xiangyong

**Affiliations:** Affiliated Renhe Hospital of China Three Gorges University, No. 410, Yiling Avenue, Yichang, 443001 China

**Keywords:** Niraparib, Anti-GD2 antibody, Osteosarcoma cells

## Abstract

**Purpose:**

This study aims to investigate the effect of Niraparib in combination with an Anti-GD2 Antibody on osteosarcoma cells.

**Methods:**

Scratch test was utilized to assess cell migration capacity, while the Transwell experiment was utilized to evaluate cell invasion potential. Cell proliferation was measured using the CCK8 experiment. The affinity between the anti-GD2 antibody and its antigen was determined via ELISA. Tumor growth was evaluated through animal experiments. Western blotting, QRT-PCR, and histological analysis were conducted to examine the expression of relevant proteins and mRNAs.

**Results:**

MG63 cell line was used for an example. The scratch test showed that the migration rate of osteosarcoma cells in Niraparib + Anti-GD2 group was 1.07 ± 0.04 after 48 h, and 0.34 ± 0.04 in the Control group. Transwell experiment showed that the invasion ability of osteosarcoma cells in Niraparib + Anti-GD2 group was 21.0 ± 1.5, and that in Control group was 87.7 ± 2.9. CCK8 experiment showed that the absorbance value of Niraparib + Anti-GD2 group was 0.16 ± 0.10 on day 5, and that of the Control group was 0.76 ± 0.09. Western blotting showed that the expression levels of BALP and CICP in Niraparib + Anti-GD2 group were 0.751 ± 0.135 and 1.086 ± 0.115, respectively, and those in Control group were 1.025 ± 0.143 and 1.216 ± 0.168, respectively. QRT-PCR results showed that the absorbance values of Niraparib + Anti-GD2 group were 0.173 ± 0.065 and 0.170 ± 0.078 on day 14. The results of animal experiments showed that on day 5, the tumor volume of the Control group was 2433 ± 391, and that of the Niraparib + Anti-GD2 group was 1137 ± 148. Histological analysis showed that the mean density values of Niraparib + Anti-GD2 group were 0.19 ± 0.08 and 0.22 ± 0.07, and those of Control group were 0.26 ± 0.09 and 0.29 ± 0.10.

**Conclusion:**

The combination of Niraparib and Anti-GD2 antibody significantly inhibits Osteosarcoma cells.

**Supplementary Information:**

The online version contains supplementary material available at 10.1007/s12672-024-01166-y.

## Introduction

Osteosarcoma (OS) is the most common primary bone malignancy, accounting for approximately 35% of all orthopedic tumors [1]. OS is a serious and destructive disease characterized by high local aggressiveness and a tendency to metastasize to the lungs and distant bones. The cure rate for OS is low, which further decreases if there are metastases. Despite rapid medical advances and various treatment options, the 5-year survival rate for patients with OS remains low at about 60–70% [2]. Therefore, it is necessary to develop new methods for treating OS patients. We focused on researching drug combination therapy for OS in hopes of improving patient survival rates.

Niraparib is a poly ADP-ribose polymerase (PARP) inhibitor that was originally developed for ovarian and breast cancer. Currently, it is primarily used for treating recurrent epithelial ovarian cancer, fallopian tube cancer, primary peritoneal cancer, and other tumor diseases. Niraparib has shown some efficacy in various tumors. According to literature, PARP inhibitors have also demonstrated a certain effect on osteosarcoma. For example, Professor Daugaard provided preclinical justification for studying the potential benefits of dual PARP and HDAC inhibition in treating Ewing sarcoma through a clinical trial and offered proof-of-concept for a bifunctional single-molecule therapeutic strategy [3]. Additionally, Professor Wang proved that combining the PARP inhibitor olaparib with irradiation (X-rays or C-ions) enhanced the radiosensitivity of osteosarcoma cell lines (U2OS and K7M2) [4]. Therefore, we believe that PARP inhibitors may be effective for osteosarcoma.

Monoclonal antibodies against disialoganglioside (GD2) have been documented to independently inhibit tumor cell viability, regardless of the immune system. A recent study found high expression of GD2 in OS tissues and cell lines. Importantly, more GD2 was expressed in OS tissues at the time of disease recurrence compared to the initial biopsy [5]. These results suggest that GD2 plays a significant role in OS progression. Gangliosides are sugar-based glycolipids containing sialic acid sheath, located in microdomains outside the plasma membrane known as “synaptic sugars”. They participate in biological processes such as cell proliferation [6]. Therefore, tumor-associated gangliosides present an attractive target for immunotherapy. Although ganglioside expression is typically limited to peripheral nerves and the central nervous system in normal tissues, studies have detected their presence in sarcoma, glioma, small cell lung cancer, neuroblastoma, and various melanoma diseases [7]. Due to its distribution pattern, GD2 has been selected as a target for monoclonal antibody therapy. Early clinical trials have demonstrated certain efficacy of monoclonal antibodies against tumor-associated gangliosides [8]. These antibodies may inhibit tumor cell viability through immune mechanisms like antibody-dependent cell-mediated cytotoxicity and complement-dependent cytotoxicity [9]. However, increasing research suggests that Anti-GD2 antibodies can suppress tumor cell viability independently of the immune system [10, 11]. Furthermore, studies have shown that Anti-GD2 antibodies can dose-dependently reduce the viability of human neuroblastoma cells [12]. We anticipate similar effects from Anti-GD2 antibody treatment for OS.

In our original research, we investigated the effect of Niraparib on OS cells alone and in combination with Anti-GD2 antibody on cell viability for the first time. The activity and invasiveness of each group of OS cells were measured using multiple detection methods to explore whether Niraparib could act on OS cells alone or enhance the effect of Anti-GD2 antibody on OS.

## Materials and methods

### Animal selection

In the present study, BALB/cA Nu/Nu nude mice were selected. The nude mice were 4 to 6 weeks old and weighed between 15 and 20 g. Specific pathogen-free (SPF) conditions were maintained to minimize the risk of infection. All animal experimental protocols conducted within this study have been rigorously reviewed and approved by the Ethical Review Committee of Renhe Hospital, China Three Gorges University. Our research is conducted in strict accordance with the ethical principles outlined in the Basel Declaration and adheres to all pertinent national laws and policies. We hereby confirm that in the course of our study, conducted under the auspices of the Ethical Review Committee (ERC) of Renhe Hospital, China Three Gorges University, the maximum tumor size/burden guidelines as established by the ERC have been strictly adhered to. No animal involved in our research has exceeded the maximum tumor size/burden limit of 1.5 cm in diameter or 10% of body weight, as approved by the ERC.

### Cells lines and reagents

SYBR Green Real-Time PCR Master Mix (Xavier Biotechnology Co., LTD.), bone alkaline phosphatase (BALP), type I collagen carboxyl terminal propeptide (CICP), and GAPDH antibodies were purchased from Xavier Biotechnology Co., LTD. Experimental cell lines MG63 (ATCC) and U2OS (ATCC) were used. Culture conditions included a gas phase of air at 95% and carbon dioxide at 5%. The temperature was set to 37 ℃, with incubator humidity maintained at 70%-80%.

### Scratch test

The sterilizable instruments have all been sterilized. The ruler and marker were irradiated with UV for 30 min before the operation (on an ultra-clean table). First, use the ruler and marker to draw horizontal lines evenly behind the 6-hole plate, about every 0.5 ~ 1 cm, across each hole. Ensure that at least 5 lines pass through each hole. Add approximately 5 × 105 cells to each hole, making sure that every hole is fully covered by cells overnight. The next day, use a pipette tip to draw horizontal lines in each hole while keeping the pipette tip vertical and not tilted. Then wash each hole three times with PBS to remove any floating cells and add serum-free medium. Place it in a 37 °C incubator with 5% CO2 for cultivation. Observe and photograph under a microscope at 0, 24, and 48 h.

### Trasnswell experiment

On the day before the experiment, a tube of Matrigel Basement Membrane Matrix that had been divided was placed in the refrigerator at 4 ℃ overnight, previously stored at – 20 ℃. The Matrigel Basement Membrane Matrix was diluted at a ratio of 1:8 and coated on the upper surface of the bottom membrane in the Transwell chamber. The residual liquid from the culture plate was removed, and each well was supplemented with 50 μL of serum-free medium containing 10 g/L BSA, incubated at 37 ℃ for 30 min. The cells were digested using conventional trypsin and washed with PBS one to two times to remove any effects from serum. Then, they were resuspended in serum-free medium and adjusted to a cell density of 5 × 105 cells/mL. Next, 200 μL of cell suspension was added to the upper chamber of Transwell chamber while adding 600 μL medium containing 10% FBS to the lower chamber of a 24-well culture plate. The culture plate was placed in a CO2 incubator at 37 ℃ for 24 h. Afterward, cells were taken out, washed twice with PBS, and carefully wiped off from the upper layer of microporous membrane using cotton swabs. Subsequently, cells were fixed with a solution containing 4% paraformaldehyde in a separate well plate for about twenty minutes followed by staining them with crystal violet solution for fifteen minutes. Finally, photographs were captured under an inverted microscope where ten random fields were counted per sample and their average value used for statistical analysis.

### CCK8 experiment.

A cell suspension was inoculated into a 96-well plate (100 μL/well). The culture plate was then placed in an incubator for pre-culture for a specific period of time (37 ℃, 5% CO2). Next, 10 μL of CCK8 solution was added to each well. The culture plate was incubated in the incubator for 2 h. Finally, the absorbance at 450 nm was measured using a microplate reader.

### Enzyme-linked immunosorbent assay (ELISA)

The GD2 antigen was immobilized in the wells of an ELISA plate and treated with a blocking solution. Then, high-affinity antibodies (positive control), low-affinity or non-specific antibodies (negative control), and experimental group antibodies (GD2 antibody) were properly diluted and added to the wells. After incubation, thorough washing of the wells was conducted. Enzyme-labeled secondary antibodies were subsequently introduced, followed by color development and reaction termination. Finally, the optical density (OD) value of each well was measured to determine antibody affinity.

### Western blotting

Protein (40 µg) was extracted, separated by 10% SDS-PAGE, and transferred to polyvinylidene difluoride membranes. The membranes were blocked with 5% skimmed milk, followed by overnight incubation with primary antibodies at 4 °C. Subsequently, the membranes were incubated with horseradish peroxidase-conjugated secondary antibodies for 2 h. Blots were developed using the ECL system. The antibodies used in our study included Anti-BALP antibody and Anti-CICP antibody.

### Real-time quantitative reverse transcription

Total RNA was extracted using the TRIzol method, and cDNA was synthesized with the RevertAidTM first-strand cDNA synthesis kit. Real-time polymerase chain reaction (PCR) was performed using the ABI PRISM 7900HT sequence detection system. The expression of osteogenesis-related genes was normalized to GAPDH using the 2−ΔΔCT method. The primer sequences are listed in Table 1.

**Table 1:** Primer sequences used in quantitative PCR assay

Gene	Sequence(5′-3′)
BALP	Forward primer: CAGAAGTGCGAGGAGGAGGT
	Reverse primer: GAAATCGTGCGGGGTCAT
CICP	Forward primer: GGTGCAGACCTAGCAGACACCA
	Reverse primer: AGGTAGCGCCGGAGTCTATTCA
GAPDH	Forward primer: GGCACAGTCAAGGCTGAGAATG
	Reverse primer: ATGGTGGTGAAGACGCCAGTA

### The establishment of the subcutaneous osteosarcoma model

The BALB/cA Nu/Nu nude mice were selected and randomly allocated into four groups: the Control group, Niraparib group, Anti-GD2 group, and Niraparib + Anti-GD2 combination therapy group. The osteosarcoma cell line (MG63) was prepared as a cell suspension and adjusted to an appropriate concentration before subcutaneously implantation in the nude mice to induce tumor formation. Tumor volume and health status were regularly assessed. Administration of the treatment was performed at a dose of 25 mg/kg once daily. The control group received an equivalent volume of placebo solution. The Niraparib group received Niraparib drug treatment. The Anti-GD2 group received Anti-GD2 antibodies for treatment. The Niraparib + Anti-GD2 combination therapy group received both Nirparab and Anti-GD-antbodies for combined treatment. Tumor growth data were periodically collected throughout the experiment duration of 5 days, after which the experiment was terminated to compare tumor growth among different groups, evaluate drug efficacy, perform statistical analysis, and determine significant differences.

### Histological analysis

Mouse tumors were sectioned and rinsed three times with PBS for 1 min each time. Cells were fixed with 4% paraformaldehyde for 15 min at room temperature, then air dried for 5 min and washed three times with PBS for 3 min each time. Cells were permeabilized with 0.5% Triton X–100 for 20 min at room temperature. Cells were then rinsed twice with PBS for 5 min each. Add 3% H2O2 solution in deionized water and incubate for 10 min at room temperature, then rinse with PBS. Add normal goat serum and incubate at 37 °C for 15 min without rinsing. Add primary antibody working solution and incubate overnight at 4 °C, then rinse twice with PBS for 5 min each time. Add HRP-conjugated secondary antibody working solution and incubate at 37 °C for 45 min, then rinse twice with PBS for 5 min each time. DAB substrate was added and the results were visualized under a microscope. Safranin was used as a stain, followed by tissue dehydration. Neutral resin was sealed, observed and photographed using a light microscope.

### Statistical analysis

The image processing was performed using Image J. Statistical analyses were conducted using SPSS for Windows 10. All data values were expressed as means ± SD. Comparisons of means among multiple groups were carried out using one-way ANOVA, followed by post hoc pairwise comparisons using Tukey’s tests. A two-tailed p-value less than 0.05 was considered statistically significant in this study.

## Results

### Scratch test

**Fig. 1:**
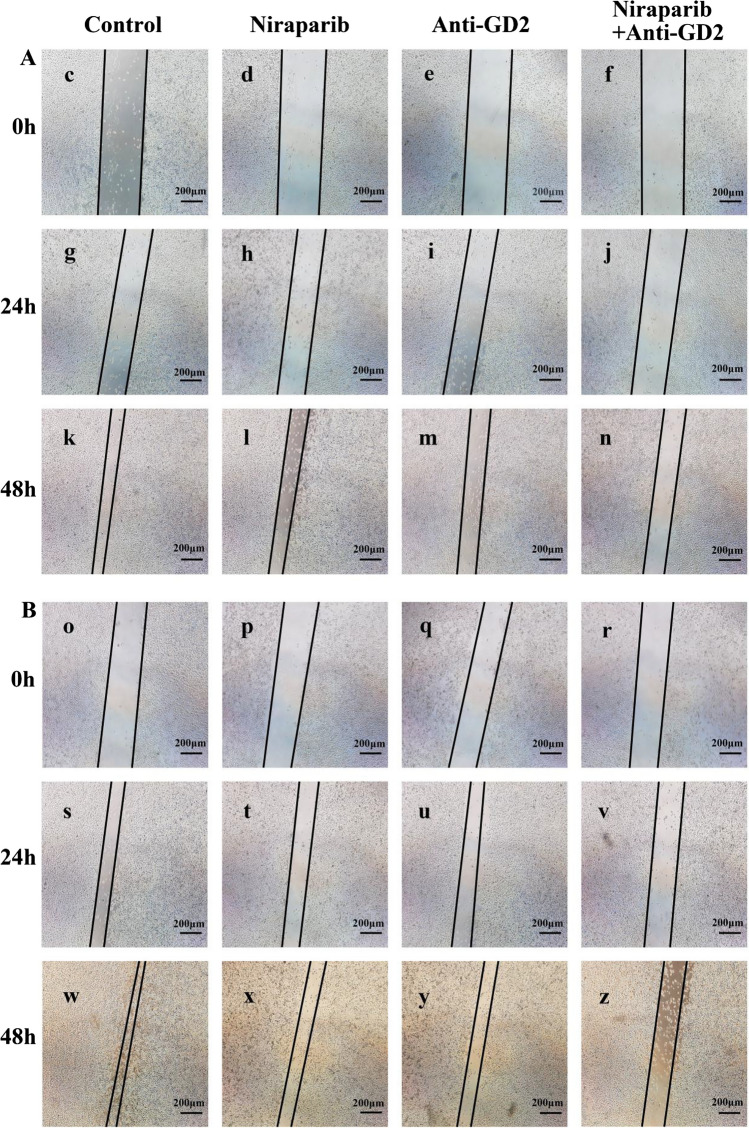
Two cell lines’ Scratch Test results of Control group, Niraparib group, Anti-GD2 group and Niraparib + Anti-GD2 group were recorded at 0 h, 24 h and 48 h, respectively. **A** MG63 cell line’s scratch test results. **B** U2OS cell line’s scratch test results. (c) MG63’s scratch test results of Control group at 0 h. (d) MG63’s scratch test results of Niraparib group at 0 h. (e) MG63’s scratch test results of GD2 group at 0 h. (f) MG63’s scratch test results of Niraparib + GD2 group at 0 h. (g) MG63’s scratch test results of Control group at 24 h. (h) MG63’s scratch test results of Niraparib group at 24 h. (i) MG63’s scratch test results of GD2 group at 24 h. (j) MG63’s scratch test results of Niraparib + GD2 group at 24 h. (k) MG63’s scratch test results of Control group at 48 h. (l) MG63’s scratch test results of Niraparib group at 48 h. (m) MG63’s scratch test results of GD2 group at 48 h. (n) MG63’s scratch test results of Niraparib + GD2 group at 48 h. (o) U2OS’s scratch test results of Control group at 0 h. (p) U2OS’s scratch test results of Niraparib group at 0 h. (q) U2OS’s scratch test results of GD2 group at 0 h. (r) U2OS’s scratch test results of Niraparib + GD2 group at 0 h. (s) U2OS’s scratch test results of Control group at 24 h. (t) U2OS’s scratch test results of Niraparib group at 24 h. (u) U2OS’s scratch test results of GD2 group at 24 h. (v) U2OS’s scratch test results of Niraparib + GD2 group at 24 h. (w) U2OS’s scratch test results of Control group at 48 h. (x) U2OS’s scratch test results of Niraparib group at 48 h. (y) U2OS’s scratch test results of GD2 group at 48 h. (z) U2OS’s scratch test results of Niraparib + GD2 group at 48 h. The experiment was repeated three times, with 24 samples each time

The scratch width did not show a significant difference between the four groups of the two cell lines at 0 h. In the MG63 cell line, the Control group’s scratch width almost disappeared at 48 h, with a distance of 0.34 ± 0.04. However, in the Niraparib + Anti-GD2 group, the scratch width remained relatively wide with a distance of 1.07 ± 0.04 (p < 0.05), and this difference was statistically significant. The U2OS cell line exhibited a similar trend as well. These results indicate that combining Niraparib with Anti-GD2 antibody has a significant inhibitory effect on OS cells’ migration (Fig. 1). Supplementary Table 1 presented the corresponding statistical data.

### Trasnswell experiment

**Fig. 2:**
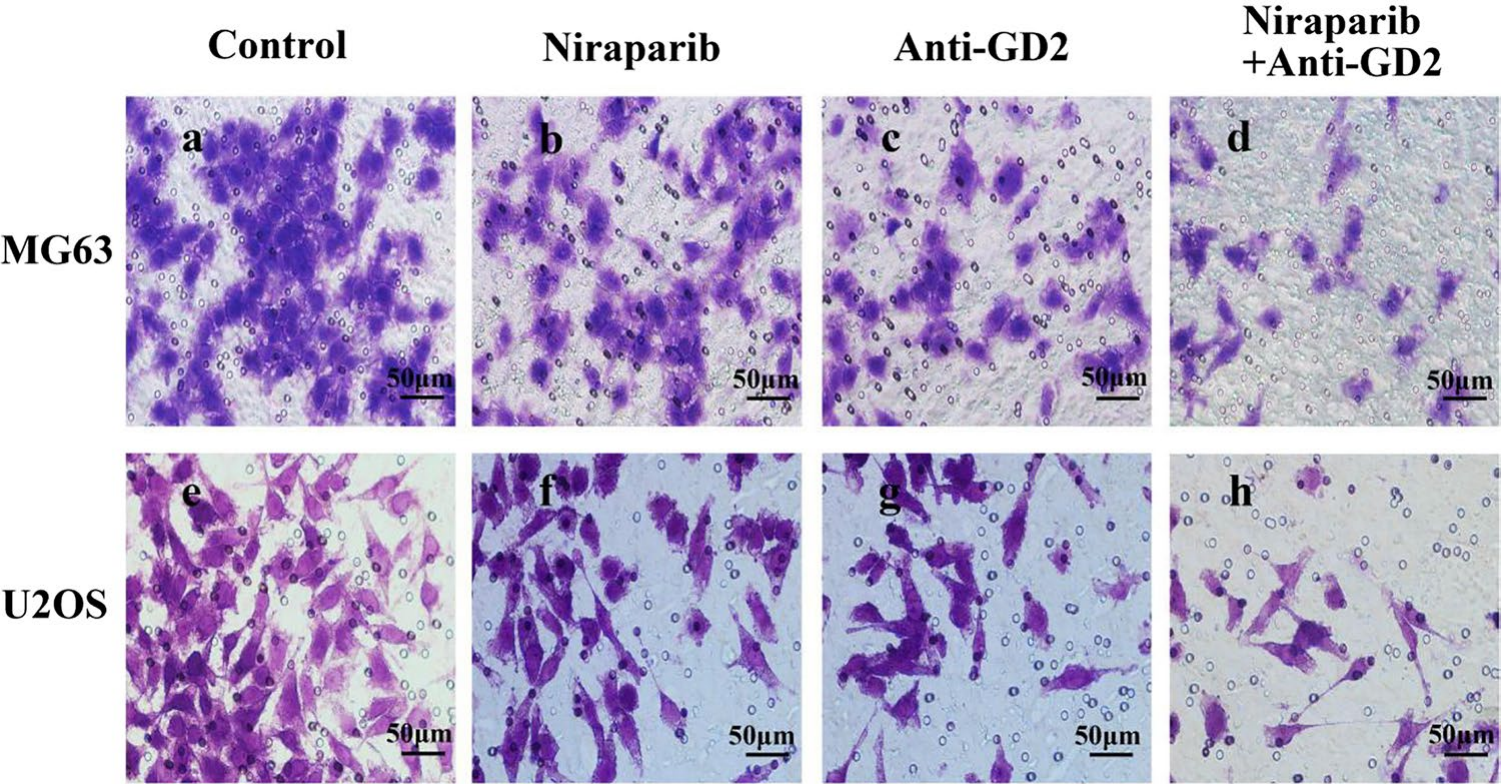
Two cell lines’ Transwell experiment results of Control group, Niraparib group, Anti-GD2 group and Niraparib + Anti-GD2 group were recorded. **a** MG63’s transwell experiment results of Control group. **b** MG63’s transwell experiment results of Niraparib group. **c** MG63’s transwell experiment results of GD2 group. **d** MG63’s transwell experiment results of Niraparib + GD2 group. **e** U2OS’s transwell experiment results of Control group. **f** U2OS’s transwell experiment results of Niraparib group. **g** U2OS’s transwell experiment results of GD2 group. **h** U2OS’s transwell experiment results of Niraparib + GD2 group. The experiment was repeated three times, with 8 samples each time

Transwell experiment results showed that in the MG63 cell line, compared with the Control group, the invasive ability of OS cells was inhibited in the Niraparib, Anti-GD2, and Niraparib + Anti-GD2 groups. However, combining Niraparib with Anti-GD2 antibody had the most significant inhibitory effect on the invasion ability of OS cells (Fig. 2). By counting, the number of cells that invaded through the Matrigel Basement Membrane Matrix in the Control group was 87.7 ± 2.9, while it was 21 ± 1.5 in the Niraparib + Anti-GD2 group (p < 0.05), indicating a statistically significant difference. The U2OS cell line exhibited a similar trend. These results demonstrate that combining Niraparib with Anti-GD2 antibody has a significant inhibitory effect on the invasive ability of OS cells. Supplementary Table 2 presented statistical data.

### CCK8 assay

**Fig. 3:**
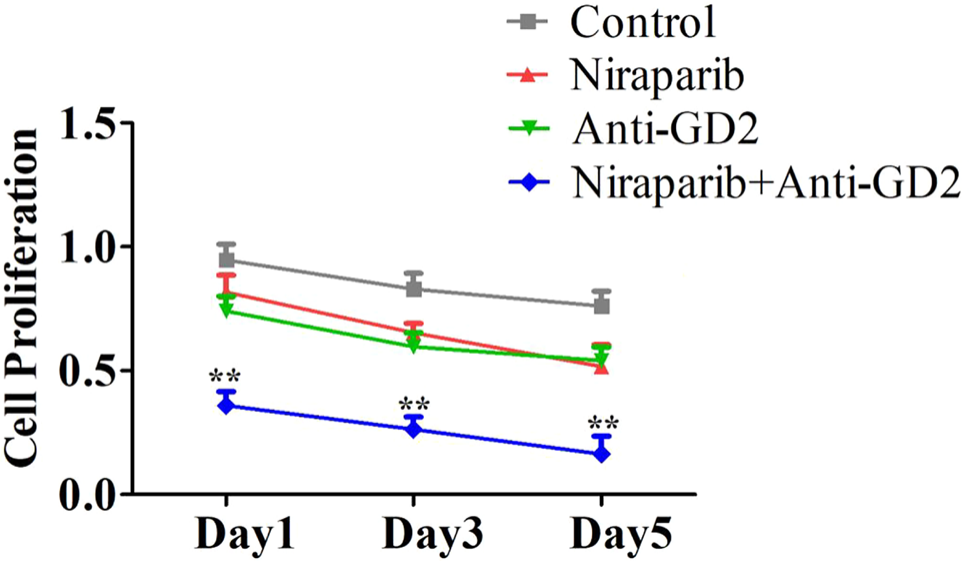
The results of the proliferation level of cells detected by CCK8 assay. The results of Control group, Niraparib group, Anti-GD2 group and Niraparib + Anti-GD2 group were recorded on day 1, 3 and 5, respectively. (gray square) Control group. (red triangle) Niraparib group. (green inverted triangle) Anti-GD2 group. (blue diamond) Niraparib + Anti-GD2. The experiment was repeated three times, with 12 samples each time

The CCK8 assay results showed that the Niraparib group and Anti-GD2 group did not have a significant inhibitory effect on OS cells’ proliferation compared to the Control group. However, when Niraparib was combined with Anti-GD2 antibody, it significantly inhibited the proliferation of OS cells (Fig. 3). As culture time increased, the inhibitory effect of Niraparib combined with Anti-GD2 antibody on OS cells’ proliferation gradually increased. On day 5, cell proliferation in the Control group was assessed by absorbance as 0.76 ± 0.09 while in the Niraparib + Anti-GD2 group it was 0.16 ± 0.10 (p < 0.05), and this difference was statistically significant. These results indicate that Niraparib combined with Anti-GD2 antibody has a significant inhibitory effect on OS cells’ proliferation. Supplementary Table 3 presented the corresponding statistical data.

### ELISA

The MG63 cell line was used as an example. After quantifying affinity based on the relative change in OD values, we observed that the positive OD value was 1.300, the negative OD value was 0.125, and the experimental group exhibited an OD value of 1.067 (Table 2). A higher OD value indicates a greater concentration of the target substance and thus signifies stronger affinity. The high-affinity antibody demonstrated significantly elevated OD values compared to its low-affinity counterpart, highlighting its superior binding capability. By comparing the ratio of OD values, we estimated that the anti-GD2 antibody possesses approximately 86.7% of the affinity displayed by the high-affinity antibody, thereby demonstrating its relatively robust binding capacity. This experimental finding effectively elucidates why anti-GD2 antibodies can still exert their functionality despite lower GD2 levels in MG63 and U2OS cells.

**Table 2:** The data of OD value in each group

Group	MG63			U2OS		
	OD value 1	OD value 2	OD value 3	OD value 1	OD value 2	OD value 3
High affinity antibodies (positive control)	1.210	1.330	1.360	1.190	1.250	1.300
Low affinity antibody (negative control)	0.100	0.150	0.120	0.120	0.120	0.160
The experimental group of GD2 antibody	0.900	1.100	1.200	1.120	1.300	0.890

### Western blotting

**Fig. 4:**
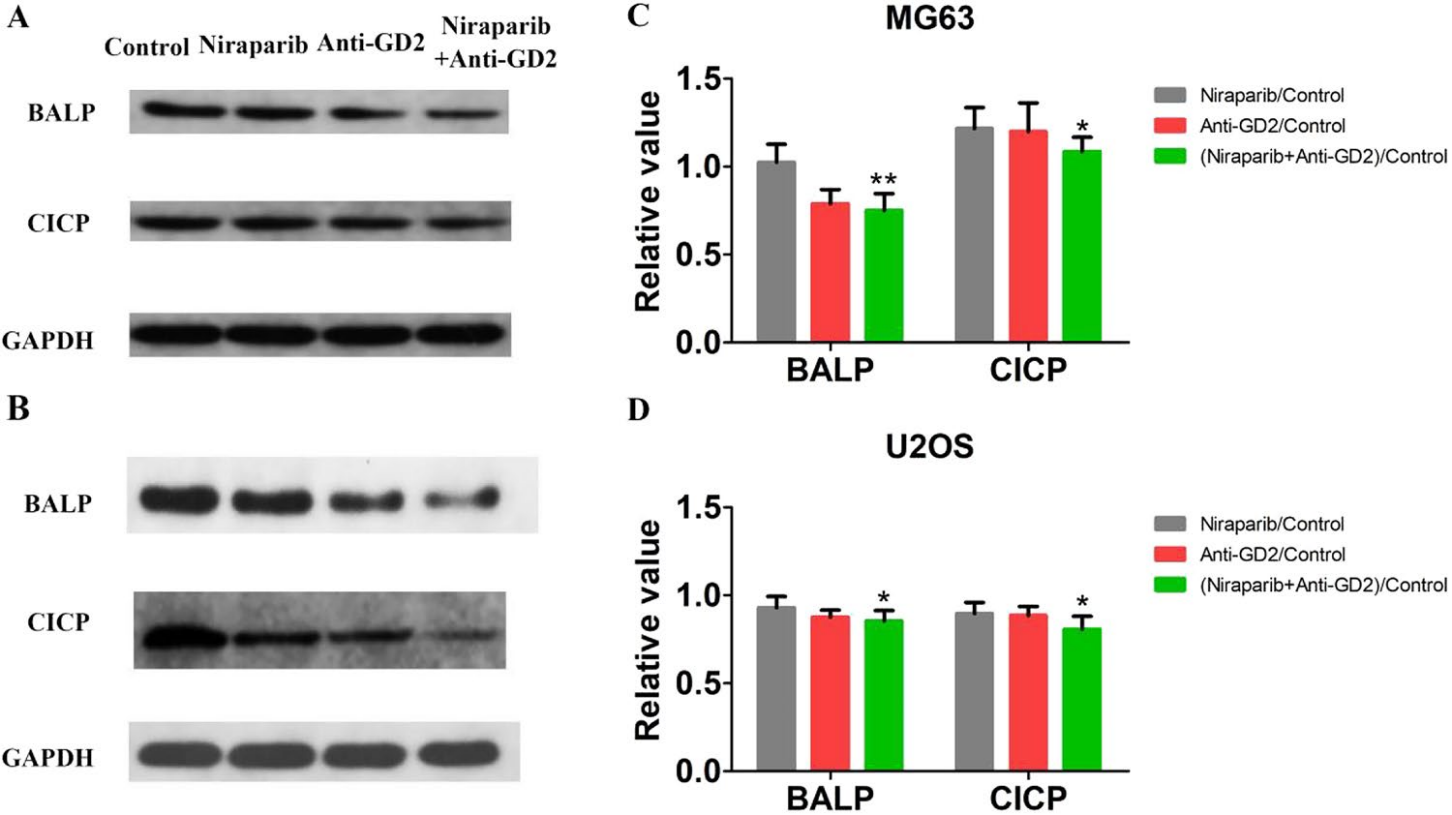
Two cell lines’ western blotting results of Control group, Niraparib group, Anti-GD2 group and Niraparib + Anti-GD2 group. The expression levels of BALP and CICP were detected with GAPDH as internal reference. **A** MG63’s western blotting results of Control group, Niraparib group, Anti-GD2 group and Niraparib + Anti-GD2 group. **B** U2OS’s western blotting results of Control group, Niraparib group, Anti-GD2 group and Niraparib + Anti-GD2 group. **C** MG63’s target protein expression of semi-quantitative analysis. (D) U2OS’s target protein expression of semi-quantitative analysis. (gray square) Relative value of Niraparib and Control group. (red square) Anti-GD2 and Control group. (green square) Niraparib + Anti-GD2 and Control group. The experiment was repeated three times, with 24 samples each time

Western blotting results showed that in the MG63 cell line, compared with the Control group, the protein expression levels of BALP and CICP were inhibited in OS cells treated with Niraparib, Anti-GD2 antibody, and Niraparib + Anti-GD2 antibody. However, the combination of Niraparib and Anti-GD2 antibody had the most significant inhibitory effect on the expression levels of BALP and CICP proteins in OS cells (Fig. 4). Through semi-quantitative analysis, the expression levels of BALP and CICP in the Control group were 1.025 ± 0.143 and 1.216 ± 0.168 respectively, while those in the Niraparib + Anti-GD2 group were 0.751 ± 0.135 and 1.086 ± 0.115 respectively (both p < 0.05), indicating statistically significant differences between groups. These results demonstrate that Niraparib combined with Anti-GD2 antibody has a significant inhibitory effect on osteogenesis-related protein expression in OS cells. Supplementary Table 4 presented the corresponding statistical data.

### Real-time quantitative reverse transcription

**Fig. 5:**
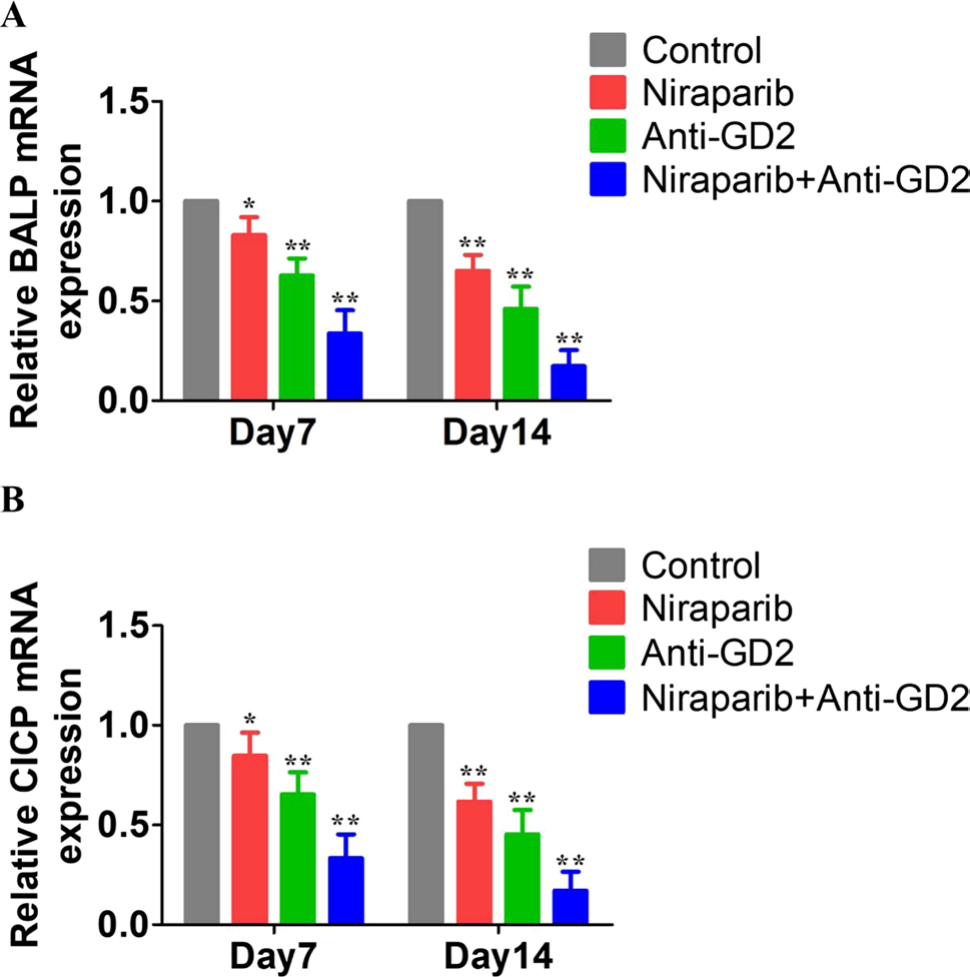
Real-time quantitative reverse transcription results of Control group, Niraparib group, Anti-GD2 group and Niraparib + Anti-GD2 group. **A** relative BALP mRNA expression. **B** relative CICP mRNA expression. (gray square) Control group. (red square) Niraparib group. (green square) Anti-GD2 group. (blue square) Niraparib + Anti-GD2 group.The experiment was repeated three times, with 16 samples each time

The PCR results showed that the mRNA expression levels of BALP and CICP were inhibited in the Niraparib group, Anti-GD2 group, and Niraparib + Anti-GD2 group compared to the Control group. However, combining Niraparib with Anti-GD2 antibody had the most significant inhibitory effect on the mRNA expression levels of BALP and CICP in OS cells. On day 14, the absorbance of mRNA expression for BALP and CICP was measured; it was 0.173 ± 0.065 and 0.170 ± 0.078 respectively for the Niraparib + AntiGD2 group compared to Control group (both p < 0.01), indicating a statistically significant difference between them. These results demonstrate that combining Niraparib with Anti-GD2 antibody has a significant inhibitory effect on osteogenesis-related RNA expression in OS cells (Fig. 5). Supplementary Table 5 presented the corresponding statistical data.

### Histological analysis

**Fig. 6:**
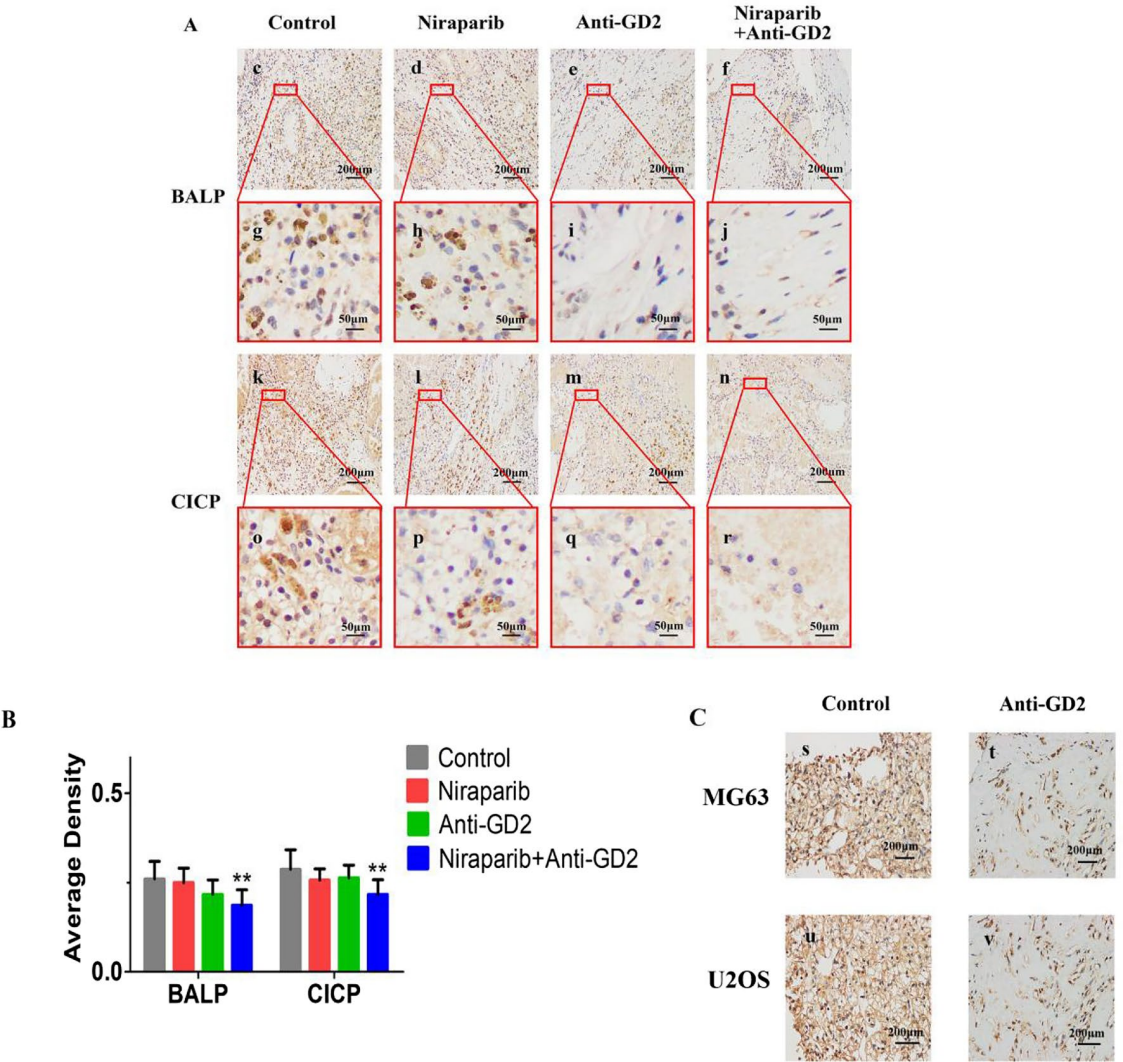
The histological analysis and average density results of BALP and CICP expression in Control group, Niraparib group, Anti-GD2 group and Niraparib + Anti-GD2 group were taken respectively. **A** histological analysis results of BALP and CICP. **B** average density results of BALP and CICP. **C** histological analysis results of GD2. (c) histological analysis result of BALP expression in Control group. (d) histological analysis result of BALP expression in Niraparib group. (e) histological analysis result of BALP expression in Anti-GD2 group. (f) histological analysis result of BALP expression in Niraparib + Anti-GD2 group. (g) partial enlarged view of BALP expression in Control group. (h) partial enlarged view of BALP expression in Niraparib group. (i) partial enlarged view of BALP expression in Anti-GD2 group. (j) partial enlarged view of BALP expression in Niraparib + Anti-GD2 group. (k) histological analysis result of CICP expression in Control group. (l) histological analysis result of CICP expression in Niraparib group. (m) histological analysis result of CICP expression in Anti-GD2 group. (n) histological analysis result of CICP expression in Niraparib + Anti-GD2 group. (o) partial enlarged view of CICP expression in Control group. (p) partial enlarged view of CICP expression in Niraparib group. (q) partial enlarged view of CICP expression in Anti-GD2 group. (r) partial enlarged view of CICP expression in Niraparib + Anti-GD2 group. (s) histological analysis results of GD2 in MG63 in Control group. (t) histological analysis results of GD2 in MG63 in Anti-GD2 group. (u) histological analysis results of GD2 in U2OS in Control group. (v) histological analysis results of GD2 in U2OS in Anti-GD2 group. (gray square) Control group. (red square) Niraparib group. (green square) Anti-GD2 group. (blue square) Niraparib + Anti-GD2 group. The experiment was repeated three times, with 8 samples each time

The histological analysis results showed that compared to the Control group, there was inhibition of BALP and CICP expression levels in OS cells observed in the Niraparib, Anti-GD2, and Niraparib + Anti-GD2 groups. However, when comparing average densities between groups (Niraparib + Anti-GD2: 0.19 ± 0.08 vs Control: 0 0.26 ± 009), statistical significance was found (p < 001). This indicates that there is a significant difference between these two groups regarding their inhibitory effects on BALP and CICP expression levels. In addition, we verified that GD2 expression was inhibited in the group with anti-GD2 antibody in both cell lines (Fig. 6).

### The establishment of the subcutaneous osteosarcoma model

**Fig. 7:**
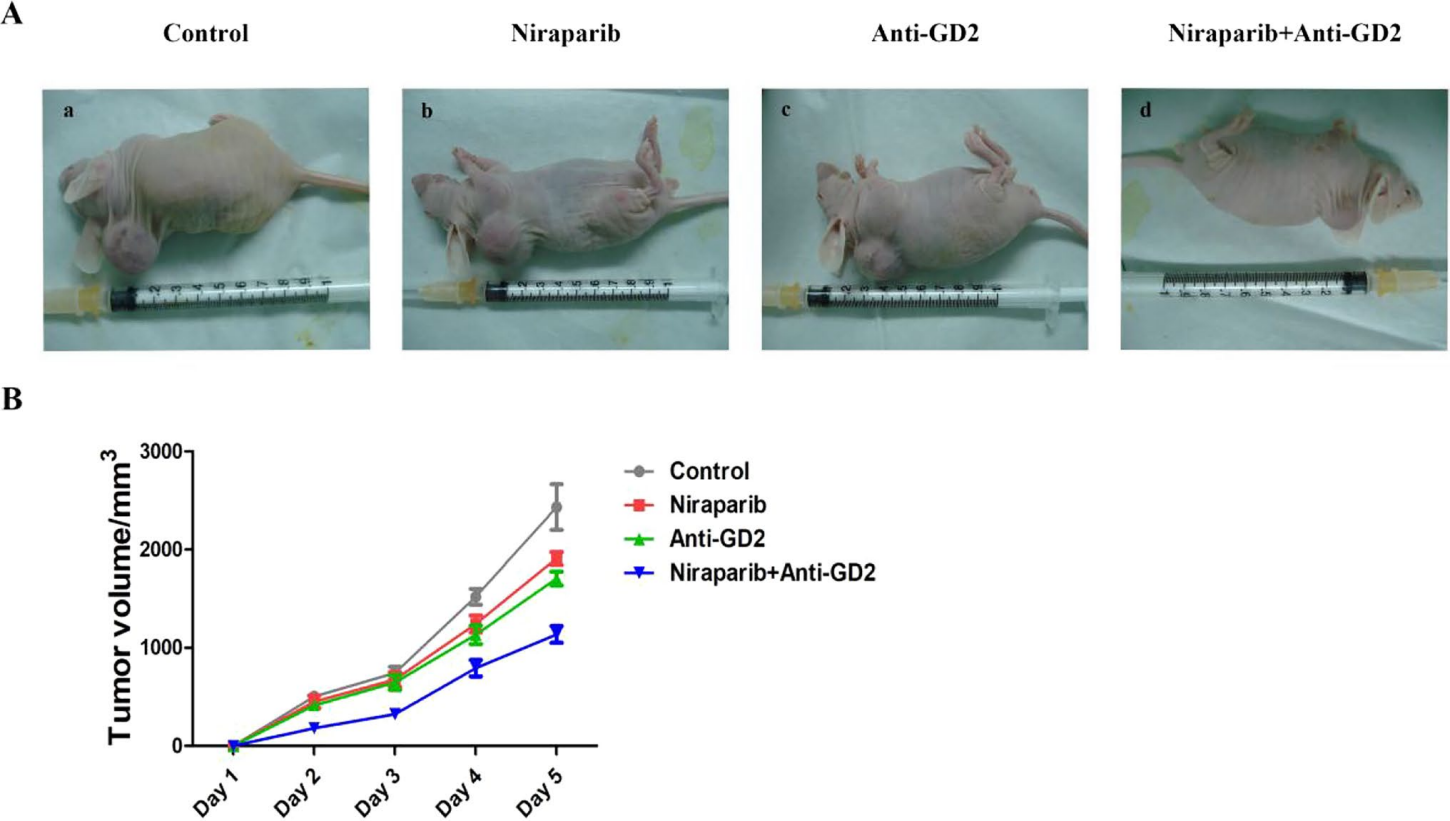
**A** Images of a subcutaneous osteosarcoma model. **B** The results of tumors volume in each group. (a) Images of a subcutaneous osteosarcoma model in Control group. (b) Images of a subcutaneous osteosarcoma model in Niraparib group. (c) Images of a subcutaneous osteosarcoma model Anti-GD2 group. (d) Images of a subcutaneous osteosarcoma model in Niraparib + Anti-GD2 group. The experiment was repeated three times, with 12 samples each time

The results of animal experiments showed that tumor growth was inhibited in the Niraparib group, Anti-GD2 group, and Niraparib + Anti-GD2 group compared to the Control group. The Niraparib + Anti-GD2 group exhibited the most pronounced inhibitory effect on tumor growth. On the fifth day, the tumor volume of the Control group was 2433 ± 391, while that of the Niraparib + Anti-GD2 group was 1137 ± 148 (p < 0.01), indicating a statistically significant difference. Combining Niraparib with an Anti-GD2 antibody demonstrated the most significant inhibition of subcutaneous tumor growth (Fig. 7).

## Discussion

The combined use of PARP inhibitors and GD antibodies has already been observed in the field of tumor treatment, and we have summarized this information in Table 3.

**Table 3:** Combined use of PARP inhibitors and GD antibodies on tumors

Author	Title	Tumor they studied	Cite
Tsao CY etc	Anti-proliferative and pro-apoptotic activity of GD2 ganglioside-specific monoclonal antibody 3F8 in human melanoma cells	Melanoma	[12]
Ha SH etc	Exogenous and Endogeneous Disialosyl Ganglioside GD1b Induces Apoptosis of MCF-7 Human Breast Cancer Cells	Breast Cancer	[13]
Durbas M etc	Downregulation of the PHLDA1 gene in IMR-32 neuroblastoma cells increases levels of Aurora A, TRKB and affects proteins involved in apoptosis and autophagy pathways	Neuroblastoma	[14]
Chung TW etc	The ganglioside GM3 is associated with cisplatin-induced apoptosis in human colon cancer cells	Colon Cancer	[15]

The results of scratch and Transwell experiments confirmed that the Anti-GD2 antibody has an effect on the invasion ability of OS cells. In the scratch test, cells in the Anti-GD2 group crawled slower than those in the Control group. In the Transwell experiment, the Anti-GD2 group showed less cell invasion compared to the Control group. Previous studies have also demonstrated a correlation between Anti-GD2 antibody and aggressive tumor cell activity [13–17], which we confirmed in OS cells.

According to preclinical studies, PARP inhibitors have potential therapeutic value for osteosarcoma (OS) [17–21]. By screening for genetic determinants of drug activity, PARP inhibitors are shown to be potential targets for OS [21–25]. The researchers also demonstrate that PARP inhibitors can enhance the activity of cytotoxic drugs [25–29]. In our study, it was confirmed by a CCK8 experiment that Niraparib had a certain effect on cell viability. Furthermore, the cell viability of the Niraparib + Anti-GD2 group was significantly reduced, indicating that Niraparib enhanced the effect of Anti-GD2 antibody.

The preclinical studies indicate that OS cells are sensitive to PARP inhibitors in vitro [29–35], but the efficacy of PARP inhibitors as monotherapy in OS is limited, highlighting the need for combination therapy. PARP inhibitors enhance DNA damage-mediated cytotoxicity caused by topoisomerase I poisons, DNA methylators, or radiation, which is relevant to the role of PARP in repairing DNA damage caused by these cytotoxic therapies [36–40]. In our study, osteogenesis was observed by detecting the expression of BALP and CICP. The results also confirmed that the effect of Niraparib monotherapy was limited. However, Niraparib could enhance the effect of Anti-GD2 antibody, and significantly reduce the expression of proteins associated with osteogenesis.

Our study emphasizes the necessity of combining PARP inhibitors with other drugs when monotherapy is ineffective in treating tumors. The study on the combination of PARP inhibitor and Anti-GD2 antibody in OS treatment demonstrated that Niraparib combined with Anti-GD2 antibody has a certain efficacy in treating OS.

It is crucial for us to emphasize this point here. We agree that subsequent treatments should be complemented by preclinical and even clinical studies. Although there are numerous potential targets for OS, our research on them remains limited. Therefore, we assert that it is premature to transition from cell experiments to clinical trials based solely on this study. The design of this study still has significant flaws, and we will gradually refine our conclusions in future research.

## Conclusion

By studying the inhibitory effect of Niraparib alone or in combination with an Anti-GD2 antibody on OS, we found that the combined treatment of Niraparib and Anti-GD2 antibody had a more prominent inhibitory effect on OS compared to Niraparib alone. This provides a novel approach for treating OS. Subsequent clinical trials will be conducted to evaluate the inhibitory effect of combining Niraparib with an Anti-GD2 antibody on OS, with the expectation of making a significant contribution to the clinical treatment of this disease.

### Supplementary Information

Supplementary Material 1.

## Data Availability

The data either reside within the article itself or can be obtained from the authors upon making a reasonable request. Contact: Wenyao Chen.

## References

[1] Yang C, Tian Y, Zhao F, Chen Z, Su P, Li Y, Qian A. Bone microenvironment and osteosarcoma metastasis. Int J Mol Sci. 2020;21(19):6985. 10.3390/ijms21196985.PMID:32977425;PMCID:PMC7582690. 32977425 10.3390/ijms21196985.PMID:32977425;PMCID:PMC7582690 PMC7582690

[2] Gill J, Gorlick R. Advancing therapy for osteosarcoma. Nat Rev Clin Oncol. 2021;18(10):609–24. 10.1038/s41571-021-00519-8. 34131316 10.1038/s41571-021-00519-8

[3] Ramos L, Truong S, Zhai B, Joshi J, Ghaidi F, Lizardo MM, Shyp T, Kung SHY, Rezakhanlou AM, Oo HZ, Adomat H, Le Bihan S, Collins C, Bacha J, Brown D, Langlands J, Shen W, Lallous N, Sorensen PH, Daugaard M. A bifunctional PARP-HDAC inhibitor with activity in ewing sarcoma. Clin Cancer Res. 2023;29(17):3541–53. 10.1158/1078-0432.CCR-22-3897. 37279093 10.1158/1078-0432.CCR-22-3897 PMC10472104

[4] Dong M, Luo H, Liu R, Zhang J, Yang Z, Wang D, Wang Y, Chen J, Ou Y, Zhang Q, Wang X. Radiosensitization of osteosarcoma cells using the PARP inhibitor olaparib combined with X-rays or carbon ions. J Cancer. 2024;15(3):699–713. 10.7150/jca.90371. 38213724 10.7150/jca.90371 PMC10777037

[5] Chen C, Xie L, Ren T, Huang Y, Xu J, Guo W. Immunotherapy for osteosarcoma: Fundamental mechanism, rationale, and recent breakthroughs. Cancer Lett. 2021;1(500):1–10. 10.1016/j.canlet.2020.12.024. 10.1016/j.canlet.2020.12.024 33359211

[6] Corre I, Verrecchia F, Crenn V, Redini F, Trichet V. The osteosarcoma microenvironment: a complex but targetable ecosystem. Cells. 2020;9(4):976. 10.3390/cells9040976. 32326444 10.3390/cells9040976 PMC7226971

[7] Meltzer PS, Helman LJ. New horizons in the treatment of osteosarcoma. N Engl J Med. 2021;385(22):2066–76. 10.1056/NEJMra2103423. 34818481 10.1056/NEJMra2103423

[8] Czarnecka AM, Synoradzki K, Firlej W, Bartnik E, Sobczuk P, Fiedorowicz M, Grieb P, Rutkowski P. Molecular biology of osteosarcoma. Cancers (Basel). 2020;12(8):2130. 10.3390/cancers12082130. 32751922 10.3390/cancers12082130 PMC7463657

[9] Smrke A, Anderson PM, Gulia A, Gennatas S, Huang PH, Jones RL. Future directions in the treatment of osteosarcoma. Cells. 2021;10(1):172. 10.3390/cells10010172. 33467756 10.3390/cells10010172 PMC7829872

[10] Zhao X, Wu Q, Gong X, Liu J, Ma Y. Osteosarcoma: a review of current and future therapeutic approaches. Biomed Eng Online. 2021;20(1):24. 10.1186/s12938-021-00860-0. 33653371 10.1186/s12938-021-00860-0 PMC7923306

[11] Zhang G, Zhao Y, Liu Z, Liu W, Wu H, Wang X, Chen Z. GD2 CAR-T cells in combination with Nivolumab exhibit enhanced antitumor efficacy. Transl Oncol. 2023;32:101663. 10.1016/j.tranon.2023.101663. 36966611 10.1016/j.tranon.2023.101663 PMC10066552

[12] González-Martín A, Pothuri B, Vergote I, DePont CR, Graybill W, Mirza MR, McCormick C, Lorusso D, Hoskins P, Freyer G, Baumann K, Jardon K, Redondo A, Moore RG, Vulsteke C, O’Cearbhaill RE, Lund B, Backes F, Barretina-Ginesta P, Haggerty AF, Rubio-Pérez MJ, Shahin MS, Mangili G, Bradley WH, Bruchim I, Sun K, Malinowska IA, Li Y, Gupta D, Monk BJ. PRIMA/ENGOT-OV26/GOG-3012 investigators. Niraparib in patients with newly diagnosed advanced ovarian cancer. N Engl J Med. 2019;381(25):2391–402. 10.1056/NEJMoa1910962. 31562799 10.1056/NEJMoa1910962

[13] Tsao CY, Sabbatino F, Cheung NK, Hsu JC, Villani V, Wang X, Ferrone S. Anti-proliferative and pro-apoptotic activity of GD2 ganglioside-specific monoclonal antibody 3F8 in human melanoma cells. Oncoimmunology. 2015;4(8): e1023975. 10.1080/2162402X.2015.1023975.PMID:26405581;PMCID:PMC4570105. 26405581 10.1080/2162402X.2015.1023975.PMID:26405581;PMCID:PMC4570105 PMC4570105

[14] Ha SH, Lee JM, Kwon KM, Kwak CH, Abekura F, Park JY, Cho SH, Lee K, Chang YC, Lee YC, Choi HJ, Chung TW, Ha KT, Chang HW, Kim CH. Exogenous and endogeneous disialosyl ganglioside GD1b induces apoptosis of MCF-7 human breast cancer cells. Int J Mol Sci. 2016;17(5):652. 10.3390/ijms17050652. 27144558 10.3390/ijms17050652 PMC4881478

[15] Durbas M, Horwacik I, Boratyn E, Rokita H. Downregulation of the PHLDA1 gene in IMR-32 neuroblastoma cells increases levels of Aurora A, TRKB and affects proteins involved in apoptosis and autophagy pathways. Int J Oncol. 2016;49(2):823–37. 10.3892/ijo.2016.3572. 27278006 10.3892/ijo.2016.3572

[16] Chung TW, Choi HJ, Kim SJ, Kwak CH, Song KH, Jin UH, Chang YC, Chang HW, Lee YC, Ha KT, Kim CH. The ganglioside GM3 is associated with cisplatin-induced apoptosis in human colon cancer cells. PLoS ONE. 2014;9(5): e92786. 10.1371/journal.pone.0092786. 24829158 10.1371/journal.pone.0092786 PMC4020741

[17] Ai X, Pan Y, Shi J, Yang N, Liu C, Zhou J, Zhang X, Dong X, He J, Li X, Chen G, Li X, Zhang H, Liao W, Zhang Y, Ma Z, Jiang L, Cui J, Hu C, Wang W, Huang C, Zhao J, Ding C, Hu X, Wang K, Gao B, Song Y, Liu X, Xiong J, Liu A, Li J, Liu Z, Li Y, Wang M, Zhang B, Zhang D, Lu S. Efficacy and safety of niraparib as maintenance treatment in patients with extensive-stage SCLC after first-line chemotherapy: a randomized, double-blind, phase 3 study. J Thorac Oncol. 2021;16(8):1403–14. 10.1016/j.jtho.2021.04.001. 33915252 10.1016/j.jtho.2021.04.001

[18] Turner NC, Balmaña J, Poncet C, Goulioti T, Tryfonidis K, Honkoop AH, Zoppoli G, Razis E, Johannsson OT, Colleoni M, Tutt AN, Audeh W, Ignatiadis M, Mailliez A, Trédan O, Musolino A, Vuylsteke P, Juan-Fita MJ, Macpherson IRJ, Kaufman B, Manso L, Goldstein LJ, Ellard SL, Láng I, Jen KY, Adam V, Litière S, Erban J, Cameron DA. BRAVO steering committee and the BRAVO investigators. Niraparib for advanced breast cancer with germline BRCA1 and BRCA2 mutations: the EORTC 1307-BCG/BIG5–13/TESARO PR-30–50–10-C BRAVO Study. Clin Cancer Res. 2021;27(20):5482–91. 10.1158/1078-0432.CCR-21-0310. 34301749 10.1158/1078-0432.CCR-21-0310 PMC8530899

[19] Wu XH, Zhu JQ, Yin RT, Yang JX, Liu JH, Wang J, Wu LY, Liu ZL, Gao YN, Wang DB, Lou G, Yang HY, Zhou Q, Kong BH, Huang Y, Chen LP, Li GL, An RF, Wang K, Zhang Y, Yan XJ, Lu X, Lu WG, Hao M, Wang L, Cui H, Chen QH, Abulizi G, Huang XH, Tian XF, Wen H, Zhang C, Hou JM, Mirza MR. Niraparib maintenance therapy in patients with platinum-sensitive recurrent ovarian cancer using an individualized starting dose (NORA): a randomized, double-blind, placebo-controlled phase III trial☆. Ann Oncol. 2021;32(4):512–21. 10.1016/j.annonc.2020.12.018. 33453391 10.1016/j.annonc.2020.12.018

[20] Lee A. Niraparib: a review in first-line maintenance therapy in advanced ovarian cancer. Target Oncol. 2021;16(6):839–45. 10.1007/s11523-021-00841-2. 34635996 10.1007/s11523-021-00841-2 PMC8613118

[21] Konstantinopoulos PA, Waggoner S, Vidal GA, Mita M, Moroney JW, Holloway R, Van Le L, Sachdev JC, Chapman-Davis E, Colon-Otero G, Penson RT, Matulonis UA, Kim YB, Moore KN, Swisher EM, Färkkilä A, D’Andrea A, Stringer-Reasor E, Wang J, Buerstatte N, Arora S, Graham JR, Bobilev D, Dezube BJ, Munster P. Single-arm phases 1 and 2 trial of niraparib in combination with pembrolizumab in patients with recurrent platinum-resistant ovarian carcinoma. JAMA Oncol. 2019;5(8):1141–9. 10.1001/jamaoncol.2019.1048. 31194228 10.1001/jamaoncol.2019.1048 PMC6567832

[22] Mirza MR, Åvall Lundqvist E, Birrer MJ, dePont Christensen R, Nyvang GB, Malander S, Anttila M, Werner TL, Lund B, Lindahl G, Hietanen S, Peen U, Dimoula M, Roed H, Ør Knudsen A, Staff S, Krog Vistisen A, Bjørge L, Mäenpää JU. AVANOVA investigators. Niraparib plus bevacizumab versus niraparib alone for platinum-sensitive recurrent ovarian cancer (NSGO-AVANOVA2/ENGOT-ov24): a randomised, phase 2, superiority trial. Lancet Oncol. 2019;20(10):1409–19. 10.1016/S1470-2045(19)30515-7. 31474354 10.1016/S1470-2045(19)30515-7

[23] Del Campo JM, Matulonis UA, Malander S, Provencher D, Mahner S, Follana P, Waters J, Berek JS, Woie K, Oza AM, Canzler U, Gil-Martin M, Lesoin A, Monk BJ, Lund B, Gilbert L, Wenham RM, Benigno B, Arora S, Hazard SJ, Mirza MR. Niraparib maintenance therapy in patients with recurrent ovarian cancer after a partial response to the last platinum-based chemotherapy in the ENGOT-OV16/NOVA Trial. J Clin Oncol. 2019;37(32):2968–73. 10.1200/JCO.18.02238. 31173551 10.1200/JCO.18.02238 PMC6839909

[24] Moore KN, Secord AA, Geller MA, Miller DS, Cloven N, Fleming GF, Wahner Hendrickson AE, Azodi M, DiSilvestro P, Oza AM, Cristea M, Berek JS, Chan JK, Rimel BJ, Matei DE, Li Y, Sun K, Luptakova K, Matulonis UA, Monk BJ. Niraparib monotherapy for late-line treatment of ovarian cancer (QUADRA): a multicentre, open-label, single-arm, phase 2 trial. Lancet Oncol. 2019;20(5):636–48. 30948273 10.1016/S1470-2045(19)30029-4

[25] Moghimi B, Muthugounder S, Jambon S, Tibbetts R, Hung L, Bassiri H, Hogarty MD, Barrett DM, Shimada H, Asgharzadeh S. Preclinical assessment of the efficacy and specificity of GD2-B7H3 SynNotch CAR-T in metastatic neuroblastoma. Nat Commun. 2021;12(1):511. 10.1038/s41467-020-20785-x. 33479234 10.1038/s41467-020-20785-x PMC7820416

[26] Park JA, Cheung NV. GD2 or HER2 targeting T cell engaging bispecific antibodies to treat osteosarcoma. J Hematol Oncol. 2020;13(1):172. 10.1186/s13045-020-01012-y.PMID:33303017;PMCID:PMC7731630. 33303017 10.1186/s13045-020-01012-y.PMID:33303017;PMCID:PMC7731630 PMC7731630

[27] Cheung IY, Cheung NV, Modak S, Mauguen A, Feng Y, Basu E, Roberts SS, Ragupathi G, Kushner BH. Survival impact of Anti-GD2 antibody response in a phase ii ganglioside vaccine trial among patients with high-risk neuroblastoma with prior disease progression. J Clin Oncol. 2021;39(3):215–26. 10.1200/JCO.20.01892. 33326254 10.1200/JCO.20.01892 PMC8253584

[28] Belayneh R, Fourman MS, Bhogal S, Weiss KR. Update on osteosarcoma. Curr Oncol Rep. 2021;23(6):71. 10.1007/s11912-021-01053-7. 33880674 10.1007/s11912-021-01053-7

[29] Markham A. Naxitamab: first approval. Drugs. 2021;81(2):291–6. 10.1007/s40265-021-01467-4. 33616889 10.1007/s40265-021-01467-4

[30] Fasanya HO, Dopico PJ, Yeager Z, Fan ZH, Siemann DW. Using a combination of gangliosides and cell surface vimentin as surface biomarkers for isolating osteosarcoma cells in microfluidic devices. J Bone Oncol. 2021;24(28): 100357. 10.1016/j.jbo.2021.100357. 10.1016/j.jbo.2021.100357 PMC8065304 33912384

[31] Bishop MW, Hutson PR, Hank JA, Sondel PM, Furman WL, Meagher MM, Navid F, Santana VM. A Phase 1 and pharmacokinetic study evaluating daily or weekly schedules of the humanized anti-GD2 antibody hu14.18K322A in recurrent/refractory solid tumors. MAbs. 2020;12(1):1773751. 10.1080/19420862.2020.1773751. 32643524 10.1080/19420862.2020.1773751 PMC7531516

[32] Chulanetra M, Morchang A, Sayour E, Eldjerou L, Milner R, Lagmay J, Cascio M, Stover B, Slayton W, Chaicumpa W, Yenchitsomanus PT, Chang LJ. GD2 chimeric antigen receptor modified T cells in synergy with sub-toxic level of doxorubicin targeting osteosarcomas. Am J Cancer Res. 2020;10(2):674–87. 32195035 PMC7061749

[33] Charan M, Dravid P, Cam M, Audino A, Gross AC, Arnold MA, Roberts RD, Cripe TP, Pertsemlidis A, Houghton PJ, Cam H. GD2-directed CAR-T cells in combination with HGF-targeted neutralizing antibody (AMG102) prevent primary tumor growth and metastasis in ewing sarcoma. Int J Cancer. 2020;146(11):3184–95. 10.1002/ijc.32743. 31621900 10.1002/ijc.32743 PMC7440656

[34] Köksal H, Müller E, Inderberg EM, Bruland Ø, Wälchli S. Treating osteosarcoma with CAR T cells. Scand J Immunol. 2019;89(3):e12741. 10.1111/sji.12741. 30549299 10.1111/sji.12741

[35] Chugh R, Ballman KV, Helman LJ, Patel S, Whelan JS, Widemann B, Lu Y, Hawkins DS, Mascarenhas L, Glod JW, Ji J, Zhang Y, Reinke D, Strauss SJ. SARC025 arms 1 and 2: A phase 1 study of the poly(ADP-ribose) polymerase inhibitor niraparib with temozolomide or irinotecan in patients with advanced Ewing sarcoma. Cancer. 2021;127(8):1301–10. 10.1002/cncr.33349. 33289920 10.1002/cncr.33349 PMC8246769

[36] He Y, Zhou X, Chen Z, Deng X, Gehring A, Ou H, Zhang L, Shi XPRAP. Pan resistome analysis pipeline. BMC Bioinform. 2020;21(1):20. 10.1186/s12859-019-3335-y.PMID:31941435;PMCID:PMC6964052. 10.1186/s12859-019-3335-y.PMID:31941435;PMCID:PMC6964052 PMC6964052 31941435

[37] Lv YX, Pan HR, Song XY, Chang QQ, Zhang DD. *Hedyotis diffusa* plus *Scutellaria barbata* suppress the growth of non-small-cell lung cancer via NLRP3/NF-*κ*B/MAPK signaling pathways. Evid Based Complement Alternat Med. 2021;2021:6666499. 10.1155/2021/6666499. 34239588 10.1155/2021/6666499 PMC8233093

[38] Wingerter A, El Malki K, Sandhoff R, Seidmann L, Wagner DC, Lehmann N, Vewinger N, Frauenknecht KBM, Sommer CJ, Traub F, Kindler T, Russo A, Otto H, Lollert A, Staatz G, Roth L, Paret C, Faber J. Exploiting gangliosides for the therapy of ewing’s sarcoma and H3K27M-mutant diffuse midline glioma. Cancers (Basel). 2021;13(3):520. 10.3390/cancers13030520. 33572900 10.3390/cancers13030520 PMC7866294

[39] Wiebel M, Kailayangiri S, Altvater B, Meltzer J, Grobe K, Kupich S, Rossig C. Surface expression of the immunotherapeutic target G_D2_ in osteosarcoma depends on cell confluency. Cancer Rep (Hoboken). 2021;4(5):e1394. 10.1002/cnr2.1394. 33811471 10.1002/cnr2.1394 PMC8551999

[40] Kailayangiri S, Altvater B, Lesch S, Balbach S, Göttlich C, Kühnemundt J, Mikesch JH, Schelhaas S, Jamitzky S, Meltzer J, Farwick N, Greune L, Fluegge M, Kerl K, Lode HN, Siebert N, Müller I, Walles H, Hartmann W, Rossig C. EZH2 inhibition in ewing sarcoma upregulates G_D2_ expression for targeting with gene-modified T cells. Mol Ther. 2019;27(5):933–46. 10.1016/j.ymthe.2019.02.014. 30879952 10.1016/j.ymthe.2019.02.014 PMC6520468

