# Supplementary material for: Combined use of niraparib enhanced the inhibitory effect of Anti-GD2 antibody on osteosarcoma cells
Source: Discov Oncol. 2024 Jul 24;15:304. doi: 10.1007/s12672-024-01166-y (PMC11269552; doi:10.1007/s12672-024-01166-y)
Supplement: Supplementary file 1 — Supplementary Material 1. [file 12672_2024_1166_MOESM1_ESM.docx]

Supplementary Materials

**Supplementary Table 1.** Scratch test

| MG63 | Control | | | Niraparib | | | GD2 | | | Niraparib+GD2 | | |
| --- | --- | --- | --- | --- | --- | --- | --- | --- | --- | --- | --- | --- |
| 0h | 1.83 | 1.92 | 1.87 | 1.96 | 2.08 | 1.89 | 1.85 | 1.93 | 1.77 | 1.88 | 1.89 | 1.93 |
| 24h | 1.17 | 1.25 | 1.33 | 1.19 | 1.26 | 1.11 | 1.24 | 1.28 | 1.20 | 1.52 | 1.46 | 1.66 |
| 48h | 0.35 | 0.29 | 0.38 | 0.63 | 0.66 | 0.81 | 0.89 | 0.99 | 0.74 | 1.01 | 1.08 | 1.11 |
| U2OS |  |  |  |  |  |  |  |  |  |  |  |  |
| 0h | 1.25 | 1.33 | 1.20 | 1.24 | 1.30 | 1.22 | 1.25 | 1.30 | 1.21 | 1.24 | 1.20 | 1.29 |
| 24h | 0.63 | 0.77 | 0.59 | 0.84 | 0.78 | 0.85 | 0.79 | 0.77 | 0.83 | 1.17 | 1.22 | 1.13 |
| 48h | 0.27 | 0.28 | 0.32 | 0.63 | 0.84 | 0.60 | 0.63 | 0.59 | 0.71 | 1.06 | 1.01 | 1.12 |

**Supplementary Table 2.** Trasnswell experiment

|  | Control | | | Niraparib | | | GD2 | | | Niraparib+GD2 | | |
| --- | --- | --- | --- | --- | --- | --- | --- | --- | --- | --- | --- | --- |
| MG63 | 84 | 91 | 88 | 59 | 52 | 62 | 43 | 46 | 38 | 20 | 17 | 26 |
| U2OS | 70 | 76 | 69 | 46 | 48 | 41 | 31 | 27 | 34 | 17 | 12 | 19 |

**Supplementary Table 3**. CCK8 experiment

|  | Control | | | Niraparib | | | GD2 | | | Niraparib+GD2 | | |
| --- | --- | --- | --- | --- | --- | --- | --- | --- | --- | --- | --- | --- |
| Day1 | 0.96 | 0.83 | 1.05 | 0.81 | 0.7 | 0.94 | 0.78 | 0.62 | 0.82 | 0.37 | 0.45 | 0.26 |
| Day3 | 0.83 | 0.94 | 0.72 | 0.65 | 0.72 | 0.59 | 0.62 | 0.49 | 0.68 | 0.28 | 0.34 | 0.17 |
| Day5 | 0.75 | 0.66 | 0.87 | 0.41 | 0.69 | 0.45 | 0.48 | 0.65 | 0.49 | 0.16 | 0.29 | 0.04 |

**Supplementary Table 4**. Western blotting

|  | relative value | Niraparib/Control | | | GD2/Control | | | (Niraparib+GD2)/Control | | |
| --- | --- | --- | --- | --- | --- | --- | --- | --- | --- | --- |
| MG63 | BALP | 0.882 | 1.221 | 0.973 | 0.674 | 0.942 | 0.753 | 0.652 | 0.942 | 0.658 |
|  | CICP | 1.077 | 1.119 | 1.453 | 1.023 | 1.523 | 1.053 | 0.990 | 1.019 | 1.248 |
| U2OS | BALP | 1.008 | 0.978 | 0.802 | 0.869 | 0.947 | 0.811 | 0.826 | 0.967 | 0.770 |
|  | CICP | 0.853 | 1.021 | 0.816 | 0.843 | 0.824 | 0.989 | 0.739 | 0.729 | 0.954 |

**Supplementary Table 5**. Real-time quantitative reverse transcription

| BALP | Control | | | Niraparib | | | GD2 | | | Niraparib+GD2 | | |
| --- | --- | --- | --- | --- | --- | --- | --- | --- | --- | --- | --- | --- |
| Day7 | 1 | 1 | 1 | 0.83 | 0.92 | 0.74 | 0.61 | 0.72 | 0.55 | 0.32 | 0.46 | 0.23 |
| Day14 | 1 | 1 | 1 | 0.65 | 0.57 | 0.73 | 0.44 | 0.58 | 0.36 | 0.18 | 0.09 | 0.25 |

| CICP | Control | | | Niraparib | | | GD2 | | | Niraparib+GD2 | | |
| --- | --- | --- | --- | --- | --- | --- | --- | --- | --- | --- | --- | --- |
| Day7 | 1 | 1 | 1 | 0.87 | 0.95 | 0.72 | 0.64 | 0.77 | 0.55 | 0.34 | 0.45 | 0.21 |
| Day14 | 1 | 1 | 1 | 0.61 | 0.53 | 0.71 | 0.48 | 0.56 | 0.32 | 0.16 | 0.08 | 0.27 |
